# Multispectral smart window: Dynamic light modulation and electromagnetic microwave shielding

**DOI:** 10.1038/s41377-024-01541-y

**Published:** 2024-08-30

**Authors:** Ruicong Zhang, Zicheng Song, Wenxin Cao, Gang Gao, Lei Yang, Yurong He, Jiecai Han, Zhibo Zhang, Tianyu Wang, Jiaqi Zhu

**Affiliations:** 1 Center for Composite Materials and Structures, Harbin Institute of Technology, Harbin, 150080 China; 2 Zhengzhou Research Institute, Harbin Institute of Technology, Zhengzhou, 450018 China; 3 Research Center of Analysis and Measurement, Harbin Institute of Technology, Harbin, 150080 China; 4 School of Energy Science & Engineering, Harbin Institute of Technology, Harbin, 150080 China

**Keywords:** Liquid crystals, Optoelectronic devices and components

## Abstract

A novel multispectral smart window has been proposed, which features dynamic modulation of light transmittance and effective shielding against electromagnetic microwave radiation. This design integrates liquid crystal dynamic scattering and dye doping techniques, enabling the dual regulation of transmittance and scattering within a single-layer smart window. Additionally, the precise control of conductive film thickness ensures the attainment of robust microwave signal shielding. We present a theoretical model for ion movement in the presence of an alternating electric field, along with a novel approach to manipulate negative dielectric constant. The proposed model successfully enables a rapid transition between light transparent, absorbing and haze states, with an optimum drive frequency adjustable to approximately 300 Hz. Furthermore, the resistive design of the conductive layer effectively mitigates microwave radiation within the 2−18 GHz range. These findings offer an innovative perspective for future advancements in environmental construction.

## Abstract (web summary)

A novel multispectral smart window achieves dynamic light modulation and robust microwave shielding.

## Graphical abstract

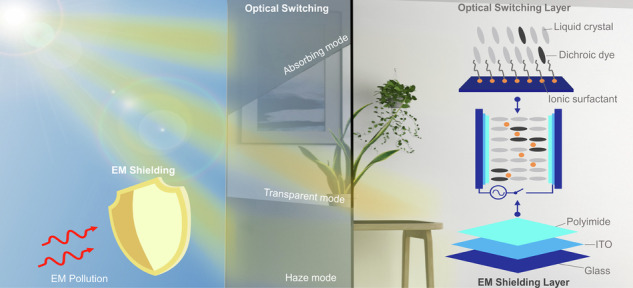

## Introduction

Windows, serving as a bridge between the external world and human living and working spaces, have a profound impact on human health and well-being. With the rapid advancement of technology, light pollution and electromagnetic (EM) pollution have emerged as significant public health concerns that cannot be ignored^1,2^. Windows, which serve as the main entry points for harmful light and EM waves into indoor spaces, need to be equipped with enhanced functionalities as a matter of urgency^3^.

Over recent decades, there has been significant progress in developing optical manipulation technologies, notably the emergence of intelligent windows capable of adaptive responses to varying environmental conditions^4–30^. For these technologies, transmittance, and haze are crucial parameters used to characterize the optical properties, as they greatly influence both performance and the overall user experience^31^. Transmittance directly affects indoor lighting intensity, consequently contributing to energy conservation. This manipulation of transmittance intensity fundamentally involves the modulation of the mediums’ visible light absorption and reflection properties, thus enabling precise management of energy flow. Conversely, haze plays a significant role in safeguarding residential privacy. It is defined as the proportion of transmitted light intensity deviating by more than 2.5° from the incident light in relation to the overall transmitted light intensity. The essence of its adjustment lies in controlling the medium’s scattering characteristic, which consequently leads to a decrease in imaging quality as haze increases.

Regrettably, the regulation of light transmission and scattering typically necessitates distinct material systems and devices to be employed. Regarding scattering regulation, traditional polymer-dispersed liquid crystal window technology primarily achieves binary or multi-state control, the rapid transformation of windows between transparent and high-haze states^9–17,32,33^. When considering transmittance adjustment, the utilization of electrochromic and thermochromic materials allows for the regulation of window transmittance. Beneath the robust transmittance control performance, these materials commonly grapple with challenges including high costs, extended transition times, and intricate processing requirements^1,4–6^. Although the integration of two material systems offers the potential for dual control of transmission and scattering, this approach can give rise to a multitude of challenges. These challenges include encompassing reduced transmittance, increased structural bulkiness, complex and expensive power supply design necessitating both alternating current (AC) and direct current (DC) sources, heightened power consumption, and disparities in response times.

Beyond dynamic modulation of visible light, the manipulation ability of EM waves is essential for the future window. With the development of the communication industry, the 5G mobile networks bring us limitless possibilities for low-latency and high-speed applications^2,34^. Nevertheless, it raises concerns regarding public health and the stability of electronic systems, attributed to the expanded frequency range, enhanced energy, and increased electromagnetic energy density^34,35^. Surprisingly, the critical aspect of electromagnetic modulation has largely been overlooked in the field of smart windows. Therefore, windows with both visible light modulation and EM shielding functions are becoming an urgent necessity.

**Fig. 1:**
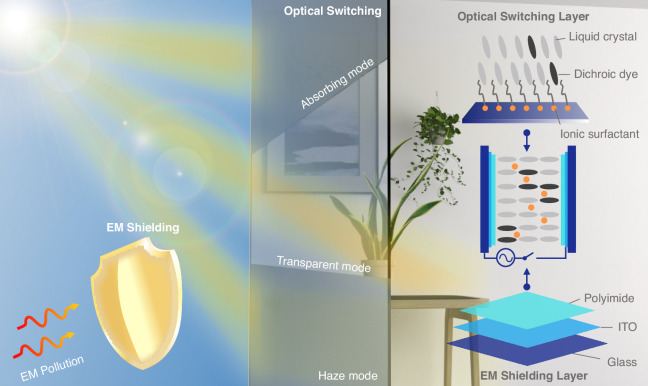
Schematic diagram of the structure and corresponding functions of the multispectral smart window in this study. The left side of the image paints a comprehensive picture of the light adjustment and electromagnetic shielding functions under a variety of conditions. These functional components work in unison to form the response system of the intelligent window, showcasing different roles based on specific needs. The right portion of the image demonstrates the structural design of the multispectral smart window

Addressing contemporary challenges and needs, we innovatively developed a multispectral smart window capable of modulating visible light and blocking microwave signals. The window’s construction, as shown in Fig. 1, includes a liquid crystal mixture core composed of negative liquid crystals, ion surfactants, and dichroic dyes, followed by vertically aligned polyimide (PI) layers and indium tin oxide (ITO) film, all encapsulated within a glass substrate. The liquid crystal mixture serves as the light modulation medium, while the ITO film functions as an electrode due to its optical transparency and conductivity, effectively blocking microwaves. We introduced a novel driving strategy combining dynamic scattering and dye doping in liquid crystal, achieving control of transmittance and scattering in a single-layer window. Precise adjustment of ITO film thickness provided robust microwave shielding, which was verified through experiments and full-wave simulations. These findings underscore our substantial progress and potential impact, with the successful development of this multispectral smart window broadening application prospects for new optical materials in light and electromagnetic modulation.

## Results

In this section, we present and assess a design strategy for a multispectral smart window aimed at achieving harmonious integration of optical dimming and microwave shielding capabilities This approach allows the visible light and microwave spectra to be designed independently, ensuring optimum dimming performance while taking electromagnetic functionality into account. The effectiveness of this design strategy demonstrates its novelty and feasibility, thereby offering a solid foundation for future optimization and implementation.

**Fig. 2:**
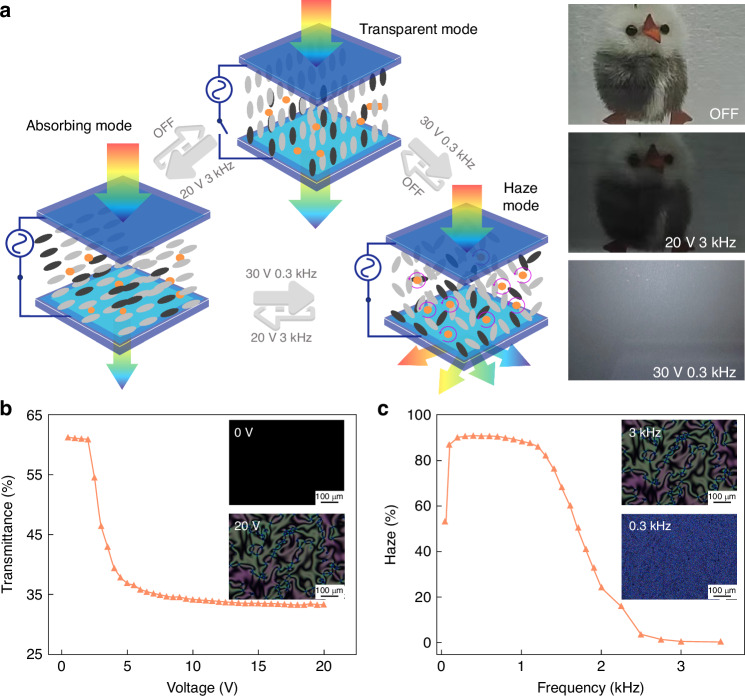
Features of light regulation in the multispectral smart window. **a** Schematic diagram of different mode dimming control strategies and effects for the tri-state switching multispectral smart window. **b** Transmittance-voltage curve and POM (Polarizing Optical Microscope) image of the tri-state switching multispectral smart window at a driving frequency of 3 kHz. **c** Haze-frequency curve and POM image of the tri-state switching multispectral smart window at a driving voltage of 30 V

In the development of the multi-mode dimming function, we manipulate the arrangement of liquid crystal molecules and dichroic dyes by precisely controlling electric field conditions to achieve different dimming effects. Figure 2a shows that without an electric field, due to the PI layer, the dichroic dye and liquid crystal molecules are vertically arranged, making the medium transparent. When a specific electric field is applied to stimulate dynamic scattering (DSM) in LCs, the continuous movement of these molecules leads to a refractive index mismatch in the dimmable dielectric layer, resulting in a haze state. On the other hand, when another specific electric field is applied, the liquid crystal molecules uniformly deflect in the direction of the electric field. As a result of the guest-host effect, the dichroic dye also deflects, which in turn regulates the medium’s transmittance, presenting an absorbing state. By flexibly controlling electric field conditions, rapid transitions between transparent, light-absorbing, and haze states can be achieved.

Simultaneously, the optimal driving frequency and threshold voltage are significant parameters for practicality and safety. Existing research on DSM indicates that the driving electric field to induce DSM must meet two fundamental prerequisites: a voltage exceeding the threshold and a frequency beneath the cutoff point^36,37^. One of the main issues with DSM’s electrical characteristics is the high operating voltage^38^. As per the predictive formula for DSM threshold voltage (Equation S1, Supporting Information), the threshold voltage is closely associated with the frequency of the applied electric field as well as the mixture’s inherent properties. In the present study, the optimal driving frequency (*f*_L_) is defined as the driving electric field frequency at the lowest threshold voltage, resulting in observing the occurrence of the DSM phenomenon. To achieve flexible regulation of voltage and frequency, we propose a method involving the introduction of ion additives into the liquid crystal mixture to alter its conductivity and dielectric constant, thus regulating its response characteristics. The findings of this research are based on the theoretical understanding of the motion state of ion additives in the liquid crystal mixture. In the discussion section of the paper, we will delve deeper into these theories and explain how ion additives affect the electrical characteristics and optical performance of the liquid crystal mixture.

The experimental results demonstrate that after the addition of 0.02 wt% sodium dodecyl sulfate (SDS) and 0.01 wt% hexadecyl trimethyl ammonium bromide (CTAB), the sample exhibits a lower optimal drive frequency and threshold voltage (Table S2, Supporting Information). To further validate our strategy, we prepare a sample containing 2 wt% nonionic dichroic dye, 0.02 wt% SDS, and 0.01 wt% CTAB and test its photoelectric performance. According to the photoelectric performance test results (refer to Fig. 2b, c), under a 3 kHz electric field, as the drive voltage increases, the transmittance of the sample decreases from 60.8% to 32.7%, reaching saturation at 10 V. Concurrently, under a drive voltage of 30 V, the haze of the sample first rises to saturation with the increase in electric field frequency and then gradually drops. This suggests that the *f*_L_ for generating DSM is 300 Hz. Additionally, in the response time test, the switching time between three different light modulation states for this device is within the range of hundreds of milliseconds (see Fig. 2a). This further confirms our hypothesis, proving that the introduction of ion additives can regulate the electrical characteristics of the liquid crystal mixture, facilitating instantaneous switching of multipartite light modulation modes. Our research provides experimental validation for the application of multipartite light modulation functionality in liquid crystal mixtures and showcases its potential application prospects.

**Fig. 3:**
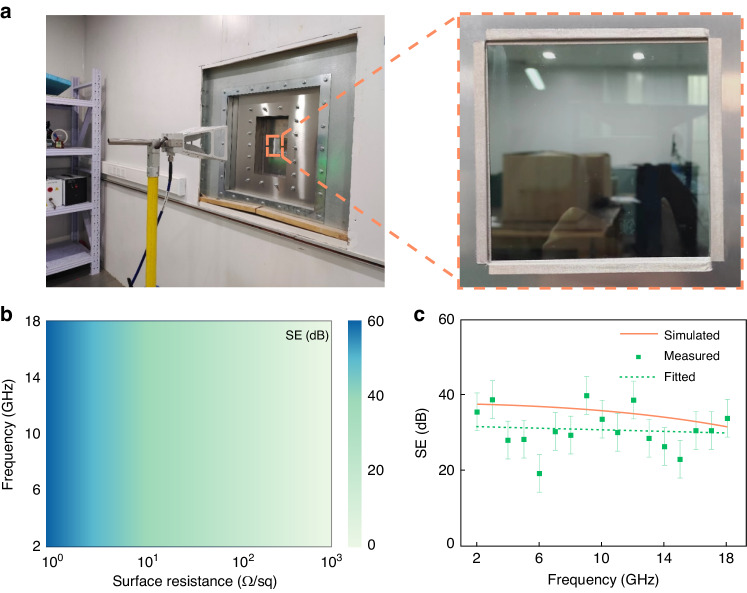
Simulated and measured results of the electromagnetic shielding performance of multispectral smart window. **a** The configuration for electromagnetic shielding experiment, and inset shows the fabricated sample. **b** The simulated shielding efficiency of the sample varies in surface resistance of the structure over the 2−18 GHz frequency range. **c** The simulated, measured, and fitted shielding effectiveness of the sample with 5 Ω sq^−1^ ITO films

To merge electromagnetic shielding performance in smart windows, we fabricate a sample and analyze it through full-wave simulations and experiments. The experimental configuration and sample pictures are shown in Fig. 3a. Through full-wave simulation, the shielding efficiency (SE) of the samples with different surface resistances in the 2−18 GHz frequency range is shown in Fig. 3b. As can be seen, the SE of the sample is effectively improved by reducing the surface resistance of the film on samples, and the broadband SE of the sample with specific surface resistance exhibits stable characteristics. The surface resistance (r) of the conductive film on the glass substrate can be defined through *r* = *ρ* / *t*, which is the resistivity (ρ) divided by the thickness of the film(t). Hence, the thickness of the film should be appropriate thicken to reduce surface resistance and enhance the broadband SE of the structure.

To validate this concept, we fabricate a 10 cm × 10 cm sample with a 350 nm-thick ITO film with a 5 Ω sq^−1^ surface resistance, which is much thicker than the usual 35 nm-thick ITO film with 80 Ω sq^−1^ surface resistance. The measurement result is shown in Fig. 3c. It can be seen that the fabricated sample achieved an average shielding effect over 30.9 dB in the 2−18 GHz range, and the highest value reached 40 dB at 10 GHz. This signifies that over 99.91% of the total incident energy is effectively shielded, and at 10 GHz, the shielded energy reaches an astonishing 99.99%. The fitted results maintain the same trend as the simulation result over broadband, while the fluctuation of the measured result is due to variations in boundary conditions and device-limited dynamic range. Overall, by flexibly adjusting the thickness of the conductive film, the proposed structure can achieve a broadband average shielding effectiveness of more than 30.9 dB.

In this section, a detailed description of the design strategies for multispectral smart windows in controlling visible light and microwave shielding is provided, along with an initial evaluation of their performance. Based on these results, the proposed method enables independent control of incident visible light and microwave signals, ensuring both dimming capability and consideration of electromagnetic performance. In the subsequent section, an in-depth study on the multi-modal regulation mechanisms adopted by multi-spectral smart windows will be conducted, followed by meticulous scrutiny. Additionally, an assessment will be made regarding the influence of these windows on the effectiveness of human protection in practical scenarios, thereby substantiating the compatibility between electromagnetic shielding and dimming performance.

## Discussion

**Fig. 4:**
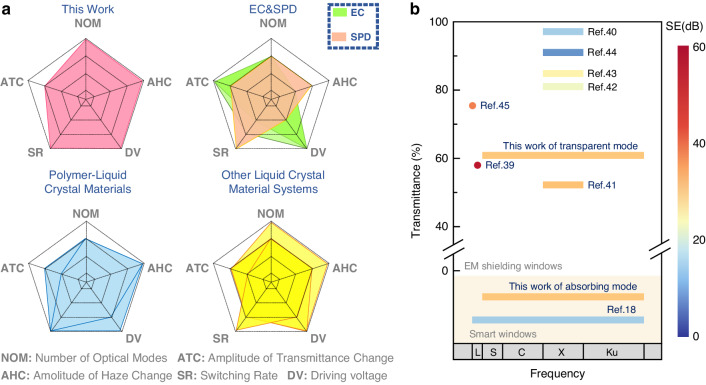
Current status analysis of multispectral smart windows. **a** Research status of electronically controlled smart windows (EC, Electrochromic smart window; SPD, suspended particle devices)^4–30^. **b** Research status of microwave shielding transparent materials^56–62^

To illustrate the advantages highlighted in this study, updated information is provided on recent advancements in electronically controlled smart window technologies and the latest developments in transparent structures for microwave shielding (Table S3, Supporting Information). The findings underscore the distinctive capabilities of the developed multispectral smart window, setting it apart from conventional structures and devices by achieving both electromagnetic shielding and optical regulation performance. As presented in Fig. 4a, across five dimensions: response time, transmittance adjustment range, haze adjustment range, driving voltage, and optical adjustment modes, this research shows a marked superiority over existing smart window technologies. For detailed analysis and evaluation criteria, see Section 5 of the supporting information. In comparison with prevalent transparent electromagnetic shielding materials, as shown in Fig. 4b, this research offers advantages in the range of covered shielding wavebands, including electromagnetic waves in the S, C, X, Ku bands. Furthermore, this approach significantly surpasses commercial PDLC (polymer dispersed liquid crystal) materials in electromagnetic shielding effectiveness. In summary, this study marks progress in merging electromagnetic shielding with optical functionalities, aiding smart window technology evolution.

**Fig. 5:**
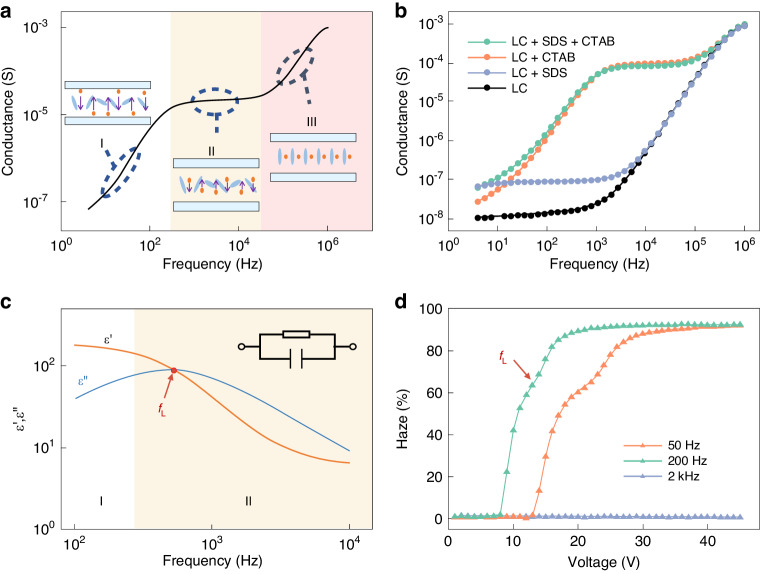
Conductance spectra. **a** Schematic diagram of the conductance spectrum of doped ion mixture. **b** Conductance spectra of samples doped with different types of ions. **c** Schematic diagram of the complex dielectric spectra of ion-doped liquid crystal mixtures. **d** Photoelectric curves of the ion-doped liquid crystal mixture samples under conditions less than the optimal driving frequency, at the optimal driving frequency and far greater than the optimal driving frequency

The foundation of our design lies in the proposed theoretical model, which is derived from our comprehension of the three potential behaviors demonstrated by ions when subjected to an alternating electric field − namely, accumulation, motion, and quiescence. As shown in Fig. 5a, in the initial phase (low-frequency accumulation region), ion migration to the liquid crystal box electrode occurs on a shorter timescale than AC field polarity changes, leading to ion accumulation at the electrodes. With increasing driving field frequency, the ion’s average movement speed rises, enhancing sample conductivity. During the second stage (mid-frequency motion region), ions exhibit continuous oscillations between the liquid crystal electrode layers. The time for ions to reach the cell electrode matches or exceeds the AC field polarity change duration. Here, the ion’s average speed peaks and sample conductivity stabilizes. In the third stage (high-frequency static region), ions remain stationary with their movement speed much slower than the AC field polarity change frequency. The system’s conductivity, influenced by the tilt angle of the liquid crystal, increases again with rising frequency.

As a proof-of-concept experiment, we measured the conductivity of four liquid crystal samples at different frequencies (Fig. 5b). The results indicate that the conductivity-frequency curves for the samples doped with the cationic surfactant CTAB conform to theoretical predictions. However, the sample containing only anionic surfactant SDS exhibited similar behavior to the sample without any ion additives. The cause of this disparity could be that the dissociated SDS^−^ is larger in volume compared to the dissociated Br^−^ from CTAB. Hence, its poorer mobility hinders its ability to keep up with the rapid polarity changes of high-frequency alternating electric fields.

In the low-frequency accumulation region, ions, given ample time, migrate to the top and bottom electrodes of the liquid crystal cell due to a low external alternating electric field frequency. This results in charge accumulation at these points, generating a counteracting electric field, attenuating the external driving field, and necessitating a higher DSM threshold voltage. In the mid-frequency motion region, ion velocity maxes out with the increase in externally applied alternating field frequency, thereby reducing their travel distance within one cycle. Within a certain frequency band, ions successfully track the up-and-down movements of the external field without any accumulation, leading to the DSM phenomenon manifesting at its lowest threshold voltage in this region. Conversely, in the high-frequency static region, overly high frequencies of the external alternating electric field prohibit ions from effectively tracking its oscillations, leaving the arrangement of the liquid crystal molecules undisturbed and rendering the DSM phenomenon unobservable. Hence, the *f*_L_ lies in the mid-frequency motion region.

To accurately determine the optimal driving field frequency, we performed further analysis using dielectric spectroscopy (Fig. 5c). According to the equivalent circuit model proposed by Belyaev et al., the real part of the complex dielectric constant ε‘ represents the capacitive characteristics of the system, while the imaginary part *ε*“ reflects its resistive characteristics^39^. When *ε*‘>*ε*“, the system primarily exhibited low-frequency capacitive characteristics, where ions could move between the poles to form an accumulation state of charge. In the mid-low frequency section where *ε*‘<*ε*“, ions adapted to changes in the external alternating field, but this process led to an increase in dielectric loss. Therefore, the optimal driving frequency of this system should be at the resonance point between the two, that is, *ε*‘=*ε*“. At this state, the current generated by the system reaches its minimum. Microscopically, ions can move freely throughout the liquid crystal pool without accumulating near the electrodes.

To validate our model, we conducted an experiment on a sample with a resonance point at 200 Hz. We tested its haze-voltage curve under 50 Hz, 200 Hz, and 2000 Hz (Fig. 5d). The test results showed that the threshold voltage significantly decreased at the *f*_L_ (200 Hz). This experimental result effectively confirmed the feasibility of our model and method, and reiterated the importance of determining the optimal driving frequency.

**Fig. 6:**
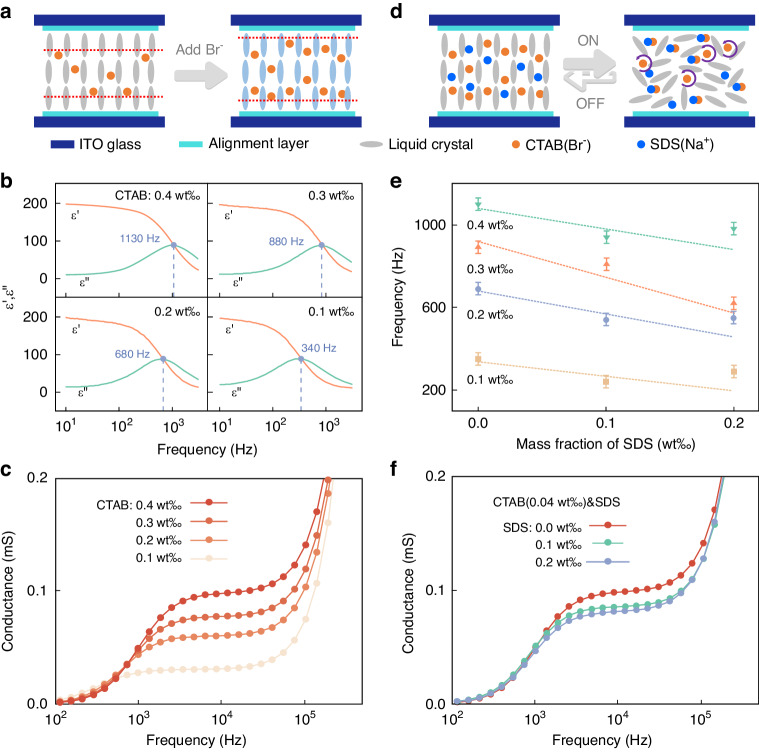
Dielectric properties of liquid crystal mixtures. **a** Schematic diagram of the structure of the liquid crystal mixture doped only with CTAB. **b** Composite dielectric spectra of liquid crystal mixtures doped with different proportions of CTAB. **c** Conductance spectrum of samples with different proportions of CTAB added. **d** Schematic diagram of the structure of the liquid crystal mixture with a co-mixing strategy of SDS and CTAB. **e** Relationship between *f*_L_ and SDS doping ratio in liquid crystal mixtures doped with different proportions of CTAB. **f** Conductance spectra of samples mixed with both CTAB and SDS

Based on these studies, it can be surmised that there is a significant positive correlation between ion concentration and *f*_L_. As Fig. 6a depicts, the shortening of average distance between ions and surface electrodes with increasing CTAB concentration indicates an augmented propensity for ion accumulation on electrodes. Therefore, a rise in CTAB concentration stimulates an elevation in *f*_L_, which is corroborated by Fig. 6b. However, under specific conditions, elevating the driving frequency could diminish the dielectric breakdown threshold of the liquid crystal^40^, necessitating a lower *f*_L_. Additionally, as manifested in Fig. 6c, the conductivity of the sample escalates correspondingly with the increase in CTAB concentration. According to Formula S1, reducing the CTAB concentration triggers an overall ascend in threshold voltage. Therefore, mere adjustments in CTAB content might lead to a predicament where the concurrent descent of threshold voltage and *f*_L_ yields counteractive effects. This suggests that more intricate strategies may be required during optimization, such as diminishing *f*_L_ while maintaining constant CTAB content.

The simultaneous inclusion of CTAB and SDS provides a viable solution to the identified issue. Upon integration into the liquid crystal matrix, SDS dissociates into negatively charged ions (SDS^−^) and Sodium ions (Na^+^). The SDS^−^ exhibits sluggish dynamics, rendering it unable to synchronize with the alternating polarity electric field. Consequently, SDS^−^ maintains homogeneous dispersion throughout the mixture, regardless of electrification status, as shown in Fig. 6d. Upon co-doping with CTAB and SDS before electrification, uniform dispersion prevails within the sample. Conversely, following electrification, Bromine ions (Br^−^) oscillate in unison with the alternating polarity electric field; however, Coulomb’s force predominantly induces their binding with omnipresent Na^+^, rather than facilitating electrode accumulation. As Fig. 6e, f indicate, co-doping of CTAB and SDS effectively diminishes *f*_L_ without sacrificing overall conductivity. This innovative approach encapsulates our projected strategy for optimizing liquid crystal mixture manipulation via complex dielectric constant modulation.

**Fig. 7:**
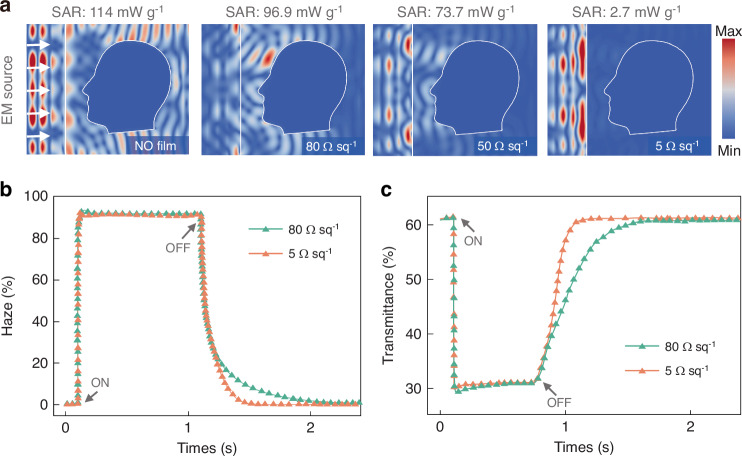
Impact of surface resistance on electromagnetic and photoelectric properties. **a** Electric field analysis of the human head behind the window with different resistance values at 5 GHz wave incidence. **b** Photoelectric response curves during the transition from transparent to absorbing states under conditions of surface resistance at 5 Ω sq^−1^ and 80 Ω sq^−1^. **c** Photoelectric response curves during the transition from transparent to haze state under conditions of surface resistance at 5 Ω sq^−1^and 80 Ω sq^−1^

Furthermore, the electric field distribution and thermal effect on human is simulated to analyze the protect effectiveness of proposed window in the practical scene. As illustrated in Fig. 7a, when transparent conductive film not loaded, window hardly influence the planewave incidence, leading to the strong field distribution around the human head. The incident electromagnetic wave can be absorbed by human body potentially affecting human health through thermal effects. The evolution of this impact is specific absorption rate (SAR), which quantifies the thermal effects is absorbed by unit mass body tissues. Under planewave irradiation with the power of 10 mW mm^−2^, the maximum SAR value among the human head is 114 mW g^−1^ without film shielding. When the conductive film is loaded, the maximum SAR is decrease to 96.9 mW g^−1^ (80 Ω sq^−1^), 73.7 mW g^−1^ (50 Ω sq^−1^), and 2.7 mW g^−1^ (5 Ω sq^−1^), respectively. This significant reduction in SAR demonstrates the effectiveness of protection, which can be visually comprehended through the E-field distribution. Most of incidence wave are reflected by the window, leading to the decrease of field amplitude around the human body. This phenomenon is significantly enhanced as the surface resistance decreases. On the other hands, the change of surface resistance does not affect the manipulation of transmittance and haze performance. Figure 7b, c compares the photoelectric response curves of the multispectral smart window to a regular smart window. It is found that the design on conductive film thickness does not negatively impact the intelligent dimming functionality, with no significant changes observed in either light transmission or response times.

In this study, we propose a novel multispectral smart window technology designed to avoids electromagnetic pollution and dynamically modulate indoor incident sunlight by transitioning between transparent, absorbing and haze states. Through fusing dynamic scattering by liquid crystals and alignment direction modulation by dichroic dyes, the proposed window can independently and rapidly modulate the transmission and scattering of visible light using a single-layer functional layer. With aid of the proposed ion motion model and dielectric constant manipulation strategy, the ratio of CTAB and SDS is confirmed to lower optimal driving frequency (around 300 Hz), thereby avoiding electrical breakdown, which is more likely to occur under high-frequency driving. Besides, the wideband electromagnetic shielding effectiveness are significantly enhanced to 30.9 dB by optimizing the thickness of transparent conductive films, while minimizing the impact on optical transparency. This study made a beneficial attempt to harmoniously design and optimize solar and microwave spectral of smart window.

The advancement of intelligent materials and communication technology is driving the transformation of smart window technologies, propelling world development towards energy efficiency and informatization. Advanced dimming functions including the manipulation of light direction and adaptive thermal adjustment in response to external conditions^41–44^, are now desirable thanks to precise liquid crystal alignment^45–47^ and innovations in stimulus-responsive materials^48–53^. By fusing reconfigurable intelligent surface technology design^54,55^, smart windows are anticipated to effectively manipulate communication signals, potentially extending wireless communication range and reducing communication delay. By fusing the solar spectrum and electromagnetic modulation, multispectral smart window technologies are shaping the future of architecture and transport.

## Materials and methods

### Materials

The compounds CTAB and SDS were procured from Macklin Inc., without undergoing further purification. The negative dielectric anisotropy liquid crystal, GXV-7002, was obtained from Yantai Xianhua Technology Group Co., Ltd. The dichroic dye (GXD-DYE800) was also sourced from Yantai Xianhua Technology Group Co., Ltd. Detailed information about these materials can be found in the supporting information. The vertically aligned LC cells were supplied by Jilin Polyrain Intelligent Technology Co., Ltd. This study utilized two types of LC cells, both with a thickness of 10μm. One type measured 20 mm × 30 mm in external dimensions, with a conductive area encompassing a circular region with a diameter of 11 mm. The other had external dimensions of 101 mm × 101 mm, with a conductive area forming a square region measuring 100 mm × 100 mm.

### Sample preparation

The LC cells were filled with the LC mixture using a capillary tube at 110 °C. The sample was slowly cooled to below the clearing point (about 95 °C) and held for 1 h to eliminate any thermal instability. Then, it was further cooled to room temperature.

### Sample characterization

The sample was examined using a polarizing optical microscope with a cross-polarizer (WMP-6880 transmission/reflection polarizing microscope, Shanghai Wumo Optical Instrument Co., Ltd.). The mid-infrared transmittance of the sample was measured using a Fourier-transform infrared spectrometer (FTIR, PE Company). The electro-optical properties of the sample were measured using a photometer (Instec, Inc. USA). The dielectric spectra of the sample in the frequency range of 4−1 × 10^6^ Hz were analyzed using an IM3536LCR meter from Hioki Corporation.

### Simulations

In the comprehensive full-wave simulations, the glass substrate exhibits a thickness of 1.1 mm alongside a relative permittivity of 5.75. The LC layer is upheld at a 10 μm thickness and a relative permittivity of 3. For the simulation concerning the infinite structure, illustrated in Fig. 3, periodic boundaries are adopted in both the x- and y-directions. For the simulation results concerning a 360 mm × 360 mm actual sample, as presented in Fig. 6, the absorbing boundaries are employed in both the x- and y-directions.

### Supplementary information

Supporting Information
