# Supplementary material for: Multispectral smart window: Dynamic light modulation and electromagnetic microwave shielding
Source: Light Sci Appl. 2024 Aug 30;13:223. doi: 10.1038/s41377-024-01541-y (PMC11362162; doi:10.1038/s41377-024-01541-y)
Supplement: Supplementary file 1 — Supporting Information [file 41377_2024_1541_MOESM1_ESM.docx]

Supplementary Information for

Multispectral Smart Window: Dynamic Light Modulation and Electromagnetic Microwave Shielding

Ruicong Zhang^1,2^, Zicheng Song^1,2^*, Wenxin Cao^1,2^, Gang Gao^1,2^, Lei Yang^3^, Yurong He^4^, Jiecai Han^5^, Zhibo Zhang^1,2^*, Tianyu Wang^4^* and Jiaqi Zhu^1,2^*

1 Center for Composite Materials and Structures, Harbin Institute of Technology, Harbin 150080, China

2 Zhengzhou Research Institute, Harbin Institute of Technology, Zhengzhou 450018, China

3 Research Center of Analysis and Measurement, Harbin Institute of Technology, Harbin 150080, China

4 School of Energy Science & Engineering, Harbin Institute of Technology, Harbin 150080, China

5 Center for Composite Materials and Structures, Harbin Institute of Technology, Harbin 150080, China

# Solubility of additives in nematic GXV-7002

To determine the solubility of additives in the nematic phase GXV-7002, a series of experiments were conducted. The figure illustrates the dispersion of different mass fractions of additives in GXV-7002. During the experiments, when the mass fraction of CTAB was 0.01 wt%, 0.02 wt%, 0.03 wt%, and 0.04 wt%, the CTAB cationic surfactant was uniformly dispersed in GXV-7002 (as shown in **Figure S1**(a)-(e)). However, when the CTAB doping concentration reached 0.05 wt%, aggregates or unmixed regions of CTAB appeared in the mixture. Similarly, when the mass fraction of SDS was 0.01 wt% and 0.02 wt%, the SDS anionic surfactant was uniformly dispersed in GXV-7002 (as shown in Figure S1(g)-(i)). However, when the SDS doping concentration reached 0.03 wt%, aggregates or unmixed regions of SDS appeared in the mixture.

Therefore, in this study, we will control the mass fraction of CTAB in the samples to not exceed 0.04 wt% and the mass fraction of SDS to not exceed 0.02 wt% to avoid interference from undissolved substances during experimental measurements. Additionally, after adding 1 wt% of the dichroic dye GXD-8002 to the nematic liquid crystal phase (as shown in Figure S1(j)), it was found to disperse uniformly. This indicates that the dichroic dye has good solubility in GXV-7002. Based on the above experimental results, we can determine the solubility of different additives in GXV-7002 and establish an appropriate doping concentration range to ensure the accuracy and reliability of experimental measurements.


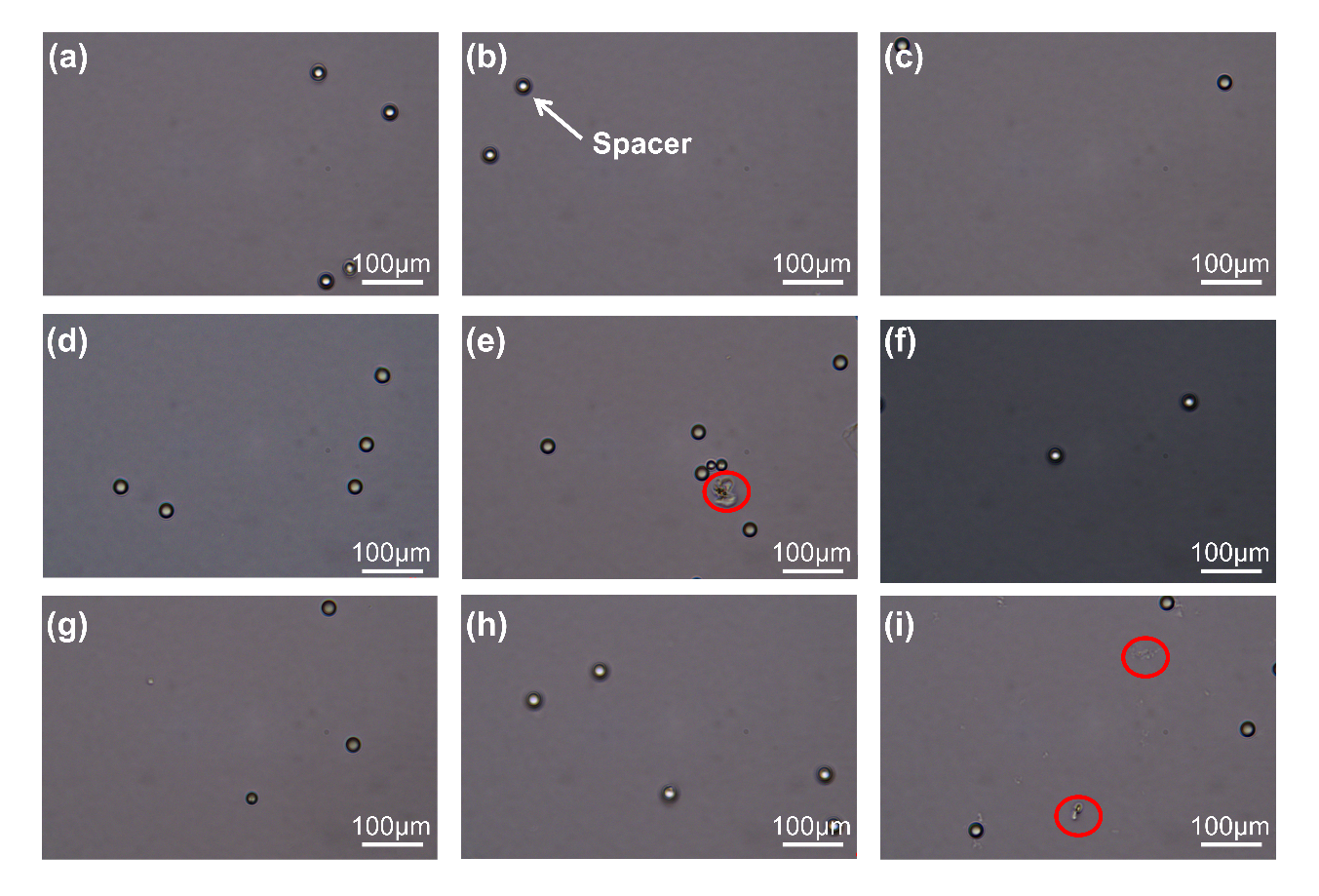


**Figure S1.** Optical microscope images of liquid crystal mixtures with different additives (red circles indicate precipitates): (a)-(e) are liquid crystal mixtures doped with CTAB at mass concentrations of 0.01 wt%, 0.02 wt%, 0.03 wt%, 0.04 wt% and 0.05 wt% respectively; (f) is a liquid crystal mixture doped with CXD-8002 dye; (g)-(i) are liquid crystal mixtures doped with CTAB at mass concentrations of 0.01 wt%, 0.02 wt% and 0.03 wt% respectively.

# Sample Preparation

The precise control of dynamic scattering is key to this study. Dynamic scattering is caused by the electrohydrodynamic instabilities (EHDI) in the current in the liquid crystal. When the electric field strength exceeds a critical value, the interaction between charges and electric fields in the liquid crystal leads to an unstable arrangement of liquid crystal molecules. The predicted formula for threshold voltage is as follows:

$V_{0}^{2}=\frac{\pi^{2}(1+\omega^{2}\tau^{2})k_{33}\sigma_{||}^{2}q^{2}}{\varepsilon_{0}^{3}\varepsilon_{||}\varepsilon_{\perp}\varepsilon_{\alpha}\text{（}\omega^{2}-\text{C）}}$ (S1)

Where,

$C=\frac{\sigma_{||}}{\varepsilon_{0}^{2}\varepsilon_{\perp}}[\frac{-\alpha_{2}}{\eta_{1}}(\frac{\sigma_{||}}{\varepsilon_{||}}-\frac{\sigma_{\alpha}}{\varepsilon_{\alpha}})-\frac{\sigma_{\perp}}{\varepsilon_{||}}]$ (S2)

*ω* is the angular frequency of the applied voltage, *τ* is the charge relaxation, *k*_33_ is the bend elastic constant, *q* is the wavenumber (approximately *π*/*d*), *σ*_⊥_ and *σ*_∥_ are the perpendicular and parallel components of the conductivity, *σ*_a_ is the conductivity anisotropy (*σ*_α_=*σ*_∥_−*σ*_⊥_), *ε*_⊥_ and *ε*_∥_ are the perpendicular and parallel components of the dielectric constants, ε_α_ is the dielectric anisotropy (*ε*_α_=*ε*_∥_−*ε*_⊥_), and *η*_1_and *α*_2_ are shear viscosities.

It can be seen that the threshold voltage of EHDI depends on the inherent mechanical properties of the mixture and is closely related to factors such as conductivity, dielectric constant, and the frequency of the applied electric field. In order to determine the optimal driving frequency (*f*_L_), we evaluated the conductivity and dielectric constant of the mixture and determined the frequency that can generate the lowest threshold voltage. Any deviation from this optimal driving frequency will result in a higher threshold voltage. The further away from the optimal driving frequency, the higher the required threshold voltage.

**Table S1.** Different doping ratios1 of various liquid crystal mixture samples.

|  | CTAB | SDS | Dye |
| --- | --- | --- | --- |
| Sample 1 | 0 | 0 | 0 |
| Sample 2 | 0.01 wt% | 0 | 0 |
| Sample 3 | 0.02 wt% | 0 | 0 |
| Sample 4 | 0.03 wt% | 0 | 0 |
| Sample 5 | 0.04 wt% | 0 | 0 |
| Sample 6 | 0 | 0.01 wt% | 0 |
| Sample 7 | 0.01 wt% | 0.01 wt% | 0 |
| Sample 8 | 0.02 wt% | 0.01 wt% | 0 |
| Sample 9 | 0.03 wt% | 0.01 wt% | 0 |
| Sample 10 | 0.04 wt% | 0.01 wt% | 0 |
| Sample 11 | 0 | 0.02 wt% | 0 |
| Sample 12 | 0.01 wt% | 0.02 wt% | 0 |
| Sample 13 | 0.02 wt% | 0.02 wt% | 0 |
| Sample 14 | 0.03 wt% | 0.02 wt% | 0 |
| Sample 15 | 0.04 wt% | 0.02 wt% | 0 |
| Sample 16 | 0.01 wt% | 0.02 wt% | 1 wt% |
| Sample 17 | 0 | 0 | 1 wt% |

To achieve a lower optimal driving frequency, we added ion additives to the liquid crystal mixture to change its conductivity and dielectric constant. Within the determined range of doping concentrations, we first prepared liquid crystal mixtures containing different types and proportions of ion surfactants, and characterized the 17 prepared samples using optoelectronic testing techniques (see **Table S1**).

# Optoelectronic Properties of Liquid Crystal Mixtures

Haze refers to the percentage of transmittance intensity deviation from the incident light at an angle greater than 2.5 degrees, which is an important indicator for characterizing a sample's ability to scatter light. In **Figure S2**, we used a photometer to explore the curve of haze change with voltage under a 50 Hz AC electric field for different doped liquid crystal mixtures at room temperature. The findings indicate that the haze of the liquid crystal blend doped with Sodium Dodecyl Sulfate (SDS) in the nematic phase, and the one devoid of additives, exhibited almost negligible alterations, thereby presenting a challenge to light scattering. This observation aligns with the previously mentioned electrical property characterizations. Conversely, for the liquid crystal blend infused with Cetyltrimethylammonium Bromide (CTAB), there was a gradual elevation in the DSM threshold voltage concurrent with escalating CTAB concentrations.


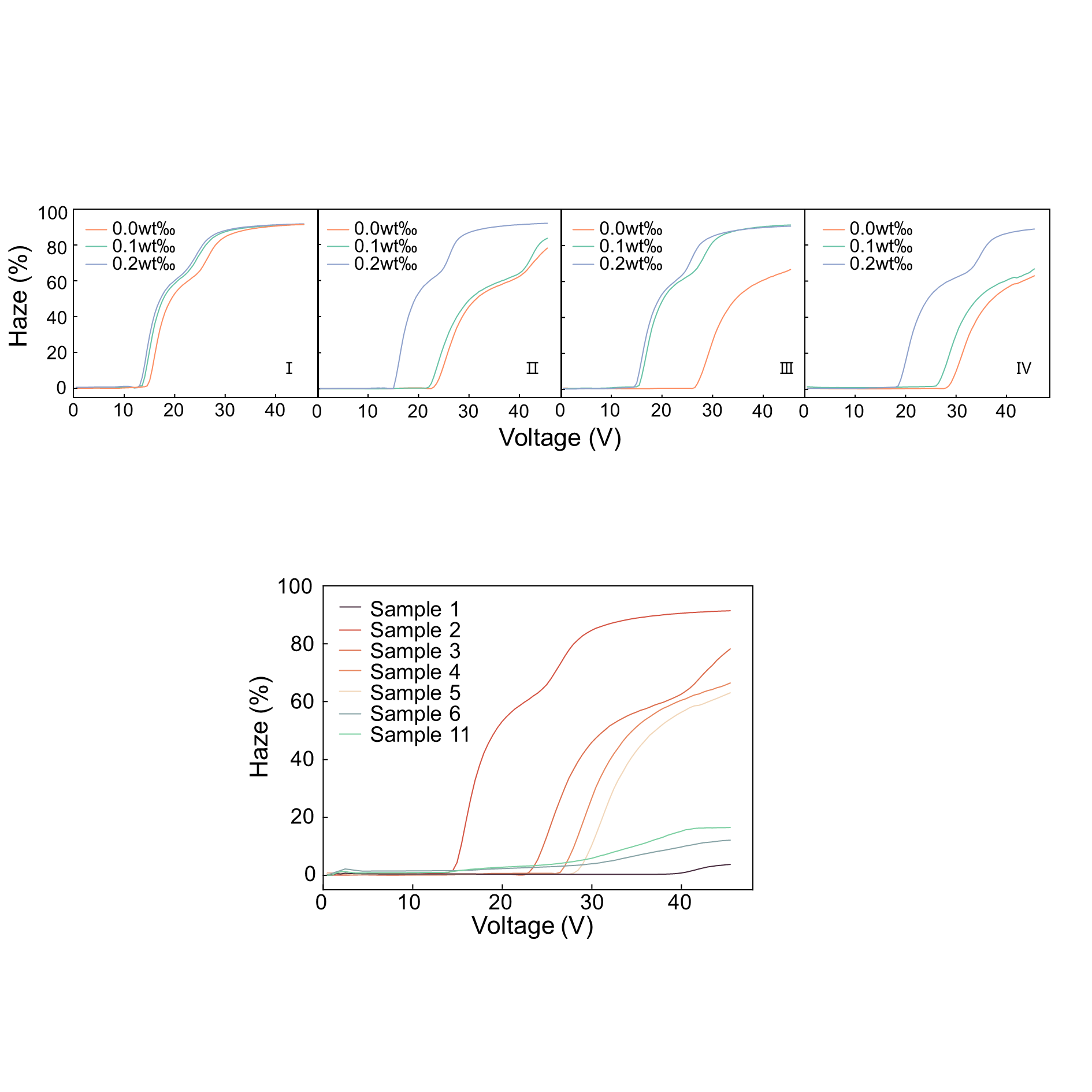


**Figure S2**. Haze-voltage curves of samples with different additives at a driving frequency of 50Hz.

From **Figure S3**, it can be seen that when CTAB and SDS are mixed, the threshold voltage of the sample decreases significantly under a 50 Hz AC electric field with an increase in SDS content. In particular, for sample 12 containing 0.01 wt% CTAB and 0.02 wt% SDS, the threshold voltage at 50 Hz drops to 13 V.


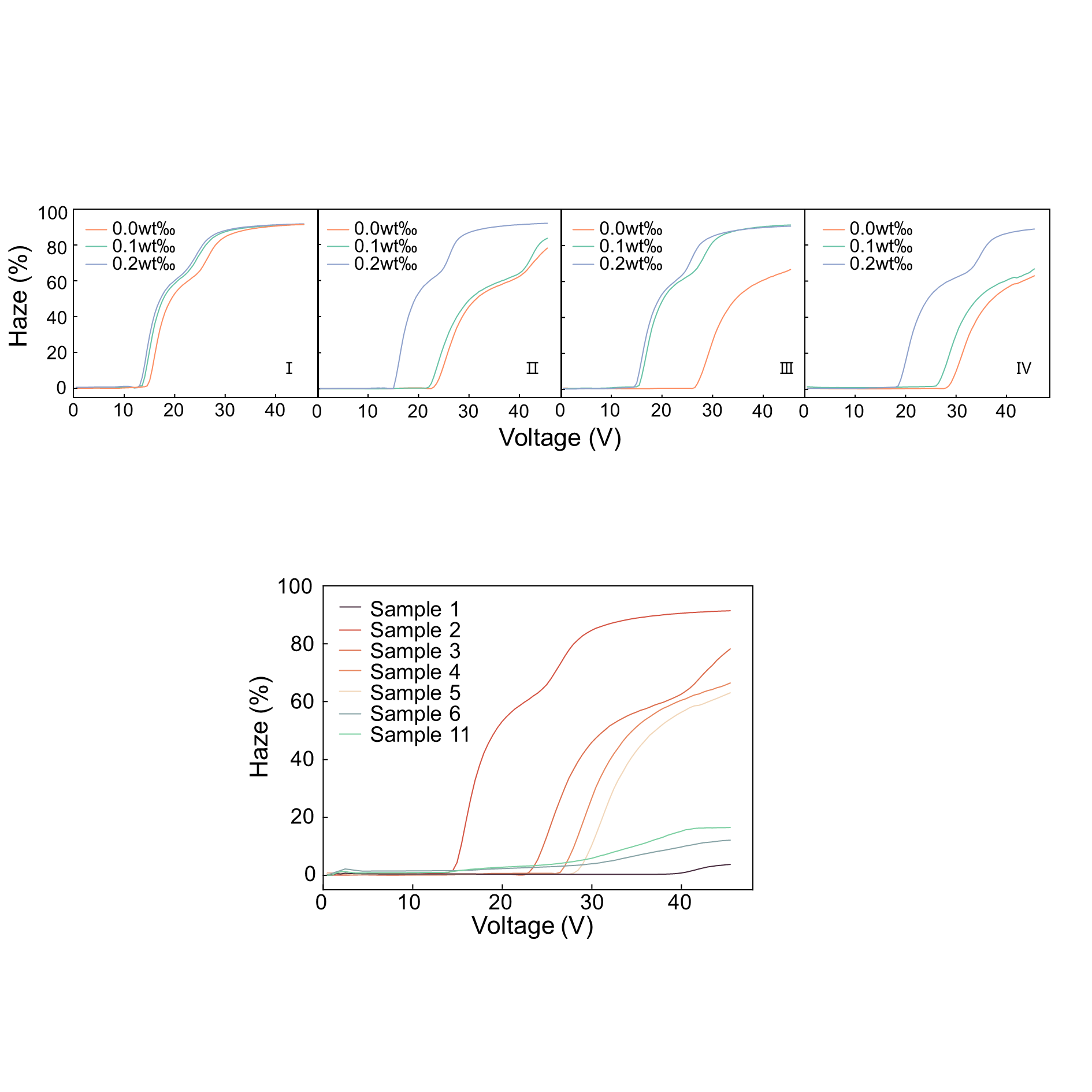


**Figure S3.** Haze-voltage curves of samples containing both CTAB and SDS at a driving frequency of 50Hz, where I, II, III, and IV represent CTAB mass concentrations of 0.01 wt%, 0.02 wt%, 0.03 wt%, and 0.04 wt%, respectively.

In **Figure S4**, the threshold voltage - driving frequency curve of the sample containing CTAB mass concentration of 0.01 wt% and SDS mass concentration of 0.02 wt% (sample 12) is shown, with driving frequencies ranging from 50Hz to 1000Hz. The curve indicates a significant decrease in threshold voltage near the optimal driving frequency of around 210 Hz.


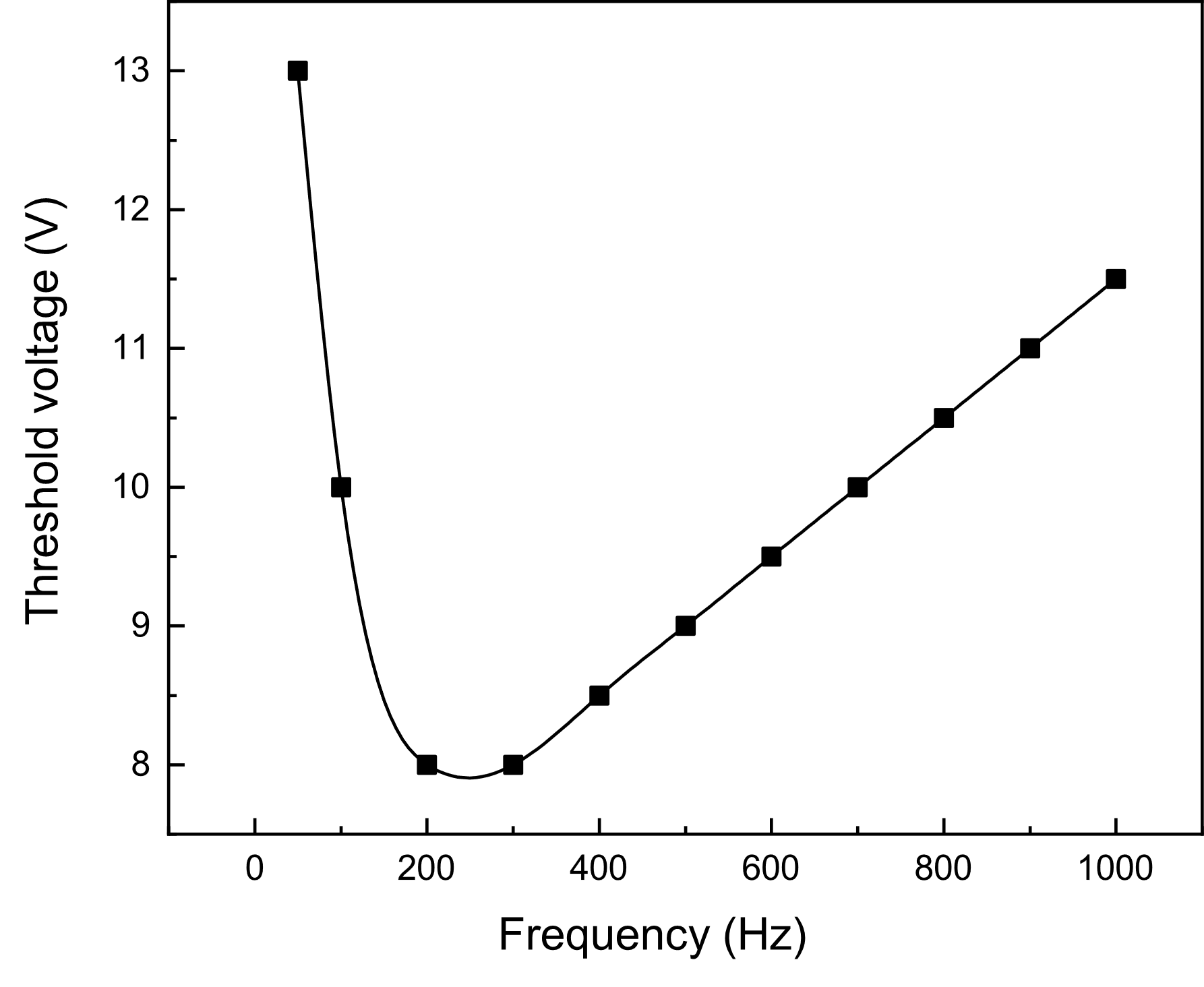


**Figure S4**. Relationship between Threshold Voltage and Driving Frequency for Samples Doped with 0.02 wt% SDS and 0.01 wt% CTAB

**Table S2.** Different doping ratios1 of various liquid crystal mixture samples.

|  | 100  Hz | 200  Hz | 300  Hz | 400 Hz | 500 Hz | 600 Hz | 700 Hz | 800 Hz | 900 Hz | 1000 Hz | *f*_L_  (Hz) |
| --- | --- | --- | --- | --- | --- | --- | --- | --- | --- | --- | --- |
| Sample 2 | 10V | 9V | 8V | 8V | 8.5V | 8.5V | 9V | 9.5V | 10V | 10.5V | 340 |
| Sample 7 | 11V | 8.5V | 8V | 8V | 8.5V | 8.5V | 9V | 9.5V | 10V | 10.5V | 230 |
| Sample 12 | 10V | 8V | 8V | 8.5V | 9V | 9.5V | 10V | 10.5V | 11V | 11.5V | 210 |
| Sample 3 | 11V | 9.5V | 9V | 8.5V | 8V | 8V | 8V | 8.5V | 9V | 9V | 680 |
| Sample 8 | 10.5V | 8.5V | 8V | 8V | 8V | 8.5V | 8.5V | 8.5V | 9V | 9V | 560 |
| Sample 13 | 11V | 8.5V | 8V | 8V | 8.5V | 8.5V | 8.5V | 8.5V | 8.5V | 9V | 480 |
| Sample 4 | 13.5V | 10V | 9V | 8.5V | 8V | 8V | 8V | 8V | 8.5V | 8.5V | 880 |
| Sample 9 | 12V | 9V | 8.5V | 8.5V | 8V | 8V | 8V | 8.5V | 8.5V | 8.5V | 790 |
| Sample 14 | 11.5V | 9V | 8.5V | 8V | 8V | 8.5V | 8.5V | 8.5V | 8.5V | 9V | 580 |
| Sample 5 | 13.5V | 11V | 9.5V | 9V | 8.5V | 8.5V | 8.5V | 8V | 8V | 8V | 1130 |
| Sample 10 | 15V | 11V | 10V | 9V | 8.5V | 8.5V | 8V | 8.5V | 8.5V | 9V | 930 |
| Sample 15 | 14V | 10.5V | 9V | 8.5V | 8.5V | 8V | 8.5V | 8.5V | 9V | 9V | 890 |

**Table S2** presents the different doping ratios of various liquid crystal mixture samples along with their corresponding threshold voltages at diverse driving frequencies. The data in the table demonstrate that these samples exhibit lower threshold voltages near the optimal driving frequency (*f*_L_), indicating the substantial impact of driving frequency on the efficient modulation of the device.

**Figure S4** displays POM images of sample 12 under different driving voltages (0V,10V,20V, and 30V) and frequencies mentioned above. Under no external field conditions, liquid crystal molecules are arranged perpendicular to the substrate and appear black; while at10 V, the POM image exhibits a typical homeotropic phase indicating that liquid crystal molecules are driven by an electric field to align parallel to the substrate direction; At 20 V, a Williams domain is observed under a frequency of 50 Hz, and liquid crystal molecules show chaotic distribution state under a frequency of 200 Hz; while still exhibiting typical homeotropic phase behavior under a frequency of 2000 Hz. At 30 V driving voltage condition, the liquid crystal molecule showed a chaotic distribution state at 50 Hz, furthermore dynamic scattering was more intense at 200 Hz, but still exhibited typical homeotropic phase behavior under a frequency of 2000 Hz. This is consistent with the aforementioned photoelectric test data.


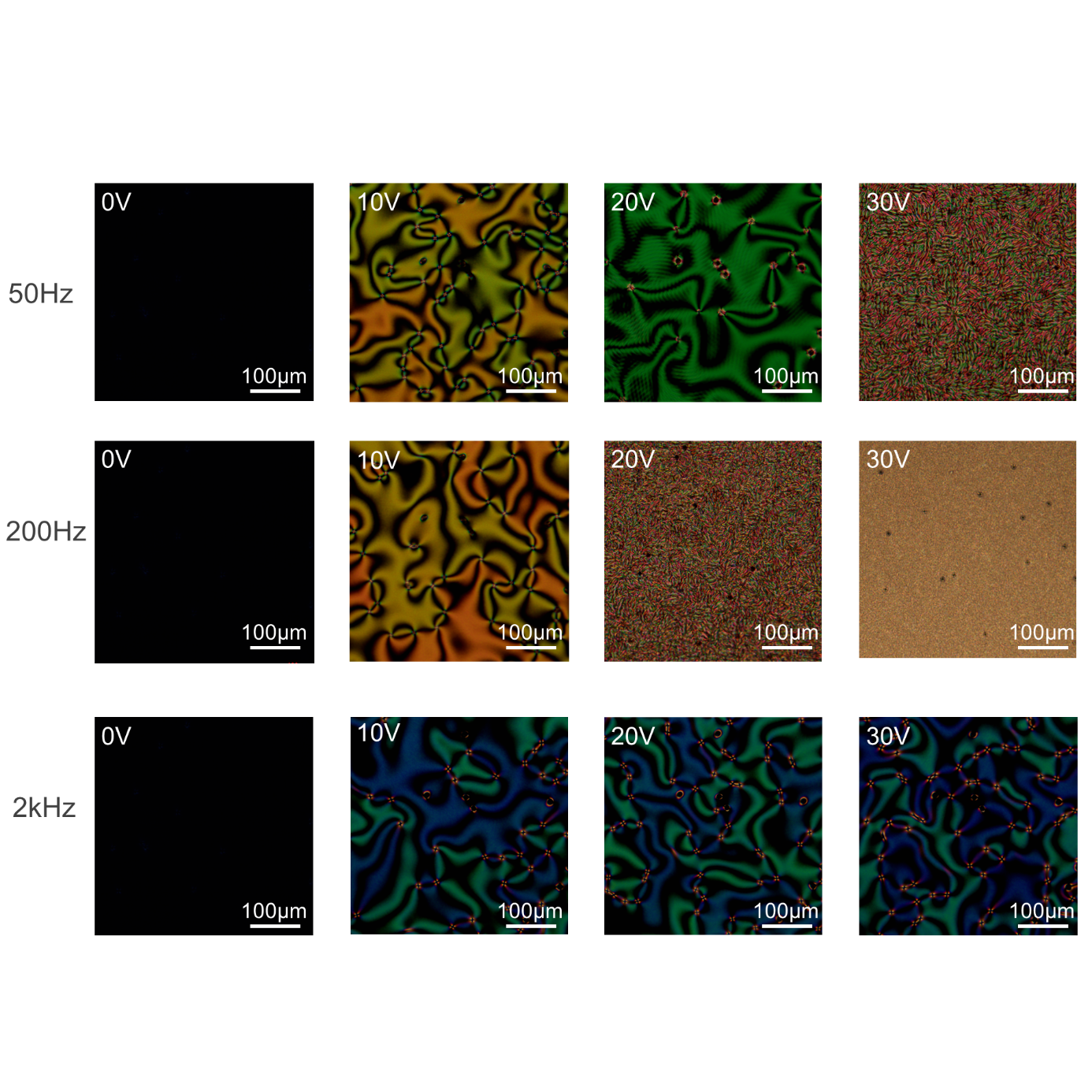


**Figure S5.** Cross-polarized optical microscope images of liquid crystal mixtures containing 0.01 wt% CTAB and 0.02 wt% SDS under different frequencies and electric fields.

# Detailed information on the use of chemical raw materials

This study investigates the dynamic scattering mode using the Electrohydrodynamic Instability (EHDI) effect, requiring the selected liquid crystal material to support fluid instability and turbulence under an electric field. This standard ensures compatibility between fluid dynamics characteristics and the EHDI effect. To effectively utilize the EHDI effect, the selected liquid crystal must exhibit negative dielectric anisotropy (Δε < 0) and positive conductivity anisotropy (Δσ > 0). These properties are crucial for achieving and maintaining the desired liquid crystal orientation under the influence of an electric field. The significant difference in refractive indices (ne and no) enhances the scattering capability of the device. As shown in **Table S3**, considering the temperature range and dielectric anisotropy, GXV-7002 was chosen as the primary material due to its excellent characteristics.

**Table S3.** Performance comparison of different types of liquid crystals

|  | clearing point  (℃) | Viscosity  (mPa.s,  20 ℃) | n_e_  (589 nm, 20 ℃) | n_o_  (589 nm, 20 ℃) | ε_\|\|_  (1 KHz,  20 ℃) | ε_┴_  (1 KHz,  20 ℃) |
| --- | --- | --- | --- | --- | --- | --- |
| GXV-7002 | 95 | 268 | 1.64 | 1.49 | 7.2 | 19.7 |

The birefringent dye GXD-DYE800 was selected based on its exceptional orientational ability, superior solubility, broad absorption band characteristics, and high photogenerated current limit. These features ensure the formation of an ordered structure within the liquid crystal system, uniform distribution, rich color rendition, while maintaining stability and efficiency. The photogenerated current performance of GXD-DYE800 shows minimal changes after long-term use, demonstrating outstanding stability and applicability, making it an ideal choice for birefringent dye selection in our research.

**Table S4.** Performance comparison of different types of dichroic dyes

|  | order parameter  (S) | Solubility (%) | | Visible light absorption properties | (μA) |
| --- | --- | --- | --- | --- | --- |
|  |  | 20 ℃ | -20 ℃ |  |  |
| GXD- DYE800 | 0.84 | ＞5 | 3.9 | Shown as **Figure S5** | 0.33 |


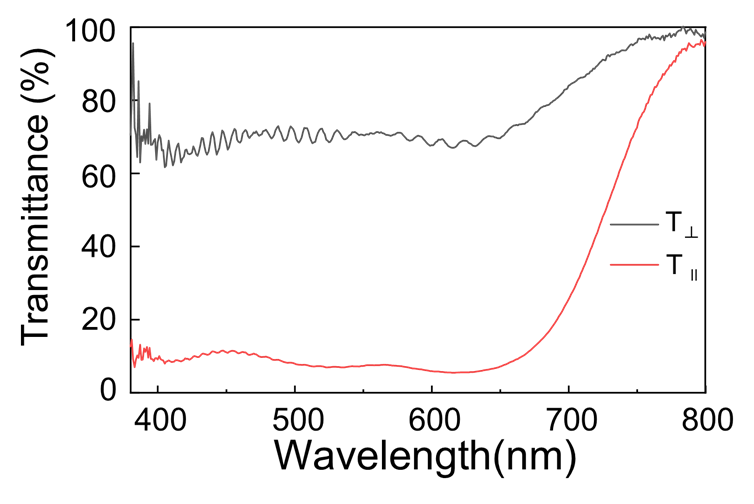


**Figure S6**. Absorption spectra of dichroic dyes


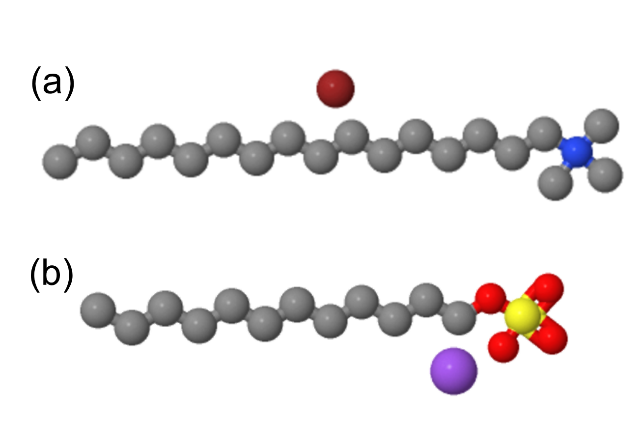


**Figure S7**. (a) Cationic surfactant CTAB (b) Anionic surfactant SDS

# Evaluation Criteria for Smart Window Technologies

**Number of Optical Modes (NOM):** This criterion assesses the versatility of smart windows in terms of their ability to switch between different visual states. A device that supports more than three distinct modes of operation, such as transparent, absorbing, and blackout, is awarded the highest score of 3, reflecting its superior adaptability to varying environmental requirements. Conversely, devices limited to two states receive a score of 2, indicative of moderate versatility.

**Amplitude of Transmittance Change (ATC):** The ability of a material to modulate light transmission is crucial for optimal performance. Materials that enable a transmittance change exceeding 50% are given a top score of 3, denoting exceptional capability in modulating light from clear to opaque transitions. Scores of 1 and 2 are assigned to materials with minor and intermediate modulation capabilities, respectively.

**Amplitude of Haze Change (AHC):** This parameter gauges a product's effectiveness in offering privacy without compromising clarity. A maximum score of 3 is allocated to products that exhibit over 80% variation in haze, ensuring significant privacy control. Products with minimal haze variation are scored at 1, with intermediate values receiving a score of 2.

**Switching Speed (SR):** Given the importance of responsiveness for user convenience, this metric evaluates the time required for a smart window to transition between states. Fast-switching devices, operating at millisecond levels, are regarded highly with a score of 3. Devices with second-level switching speeds are scored at 1, acknowledging their slower response times. Intermediate speeds are awarded a score of 2.

**Driving Voltage (DV):** Energy efficiency and safety are paramount; thus, devices operating at voltages below the human-safe threshold of 36V are given the highest score of 3. Devices necessitating up to 110V score 2 for moderate safety and efficiency, while those requiring in excess of 110 V score 1, highlighting potential safety concerns due to high operational voltages.

For detailed data supporting these evaluations, please refer to Table S3 provided in the supplementary information.

**Table S3.** Typical smart windows with adjustable optical functions and microwave shielding capabilities. Symbol "-" indicates the terms that have not been concerned or targeted in the study.

| type of window | Optical function manipulation | | | | | | Microwave shielding function | | | | | Ref. |
| --- | --- | --- | --- | --- | --- | --- | --- | --- | --- | --- | --- | --- |
|  | Number of optical modes | T_p_ | T_t_ | H | t_on_/t_off_ | Switching electric field conditions | | Electromagnetic shielding effectiveness  (dB) | | | Effective microwave shielding range(GHz) |  |
| LC | 3 | 0.61-0.33-0.01 | 0.61-0.33-0.18 | 0.0-0.1-0.89 | 12.7 ms/671.0 ms(T-D) 18.1 ms/50.2 s(D-H) 3.0 ms/359.2 ms(T-H) | 0 V-15 V,3 kHz-30 V,300 Hz | | 30.9 | 2-18 | | | This  work |
| EC | 2 | 0.99-0.21 | - | - | 0.5 s/0.2 s | -2.5 V- +2.5 V | | Not touched | | | | S1 |
| EC | 2 | 0.92-0.10 | - | - | 11.5 s/6 s | -1 V- +1V | | Not touched | | | | S2 |
| EC | 2 | 0.94-0.25 | - | - | 6.4 s/7.2 s | -1 V- +1V | | Not touched | | | | S3 |
| SPD | 2 | 0.64-0.18 | - | - | - | 0 V-110 V | | Not touched | | | | S4 |
| SPD | 2 | 0.05 -0.55 | - | - | - | 0 V-110 V | | Not touched | | | | S5 |
| P-LC | 2 | 0.07 -0.62 | 0.23-0.63 | 0.71-0.01 | - | 0 V-37 V | | Not touched | | | | S6 |
| P-LC | 2 | 0.02 -0.78 | - | - | 183 ms/- | 0 V-32.3 V | | Not touched | | | | S7 |
| P-LC | 2 | 0.05 -0.80 | - | - | 50 ms/350 ms | 0 V-24 V | | Not touched | | | | S8 |
| P-LC | 2 | 0.01 -0.65 | - | - | 40 ms/- | 0 V-50 V | | Not touched | | | | S9 |
| P-LC | 2 | 0.00 -0.78 | - | - | 55 ms/- | 0 V-55 V | | Not touched | | | | S10 |
| P-LC | 2 | 0.00 -0.65 | - | - | 100 ms/- | 0 V-34.2 V | | Not touched | | | | S11 |
| P-LC | 2 | 0.61 -0.01 | - | - | 84 ms/- | 0 V-40 V | | Not touched | | | | S12 |
| P-LC | 2 | 0.67 -0.03 | - | - | 1 ms/0.47 ms | 0 V-30 V | | Not touched | | | | S13 |
| P-LC | 2 | 0.04 -0.98 | - | - | 21 ms/6 ms | 0 V-40 V | | Not touched | | | | S14 |
| P-LC | 2 | - | - | - | - | - | | 15 | | 1-18 | | S15 |
| LC | 3 | - | 0.81-0.42-~0.1 (λ=560 nm) | ~0-~0-0.94 | 1.0 s/200 ms(T-H) | 0 V - 30 V,5 kHz - 30 V,1 kHz | | Not touched | | | | S16 |
| LC | 2 | 0.70 -0.01 | - | - | 2.5 ms/1020 ms | 0 V-60 V | | Not touched | | | | S17 |

| type of window | Optical function manipulation | | | | | | Microwave shielding function | | | Ref. |
| --- | --- | --- | --- | --- | --- | --- | --- | --- | --- | --- |
|  | Number of optical modes | T_s_ | T_t_ | H | t_on_/t_off_ | Switching electric field conditions | Electromagnetic shielding effectiveness  (dB) | Effective microwave shielding range(GHz) | |  |
| LC | 2 | 0.90 -0.16 | - | - | - | 0 V-20 V | Not touched | | | S18 |
| LC | 2 | - | 0.61-0.21 | 0.1-0.86 | - | 0 V-40 V | Not touched | | | S19 |
| LC | 3 | - | 0.64-0.21-0.21 | 0-0.1-0.72 | - | 0 V-20 V-65 V | Not touched | | | S20 |
| LC | 2 | 0.89 -0.26 | - | - | 10 ms/100 ms | 0 V-112 V | Not touched | | | S21 |
| LC | 2 | - | - | - | 6.43 ms/44.2 ms | - | Not touched | | | S22 |
| LC | 3 | 0.50-0.15-0.05 | - | 0.0-0.1-0.70 | - | 0 V -12 V -40 V | Not touched | | | S23 |
| LC | 3 | - | 0.37-0.12-0.12 | 0.12-0.14-0.79 | 2000 ms/44 ms  (T-D) 44 ms/0.13 s(D-H) 2.13 s/860 ms(T-H) | 40 V-120 V-80 V | Not touched | | | S24 |
| LC | 2 | 0.38-0.66 | - | 0.4-0.18 | 2 ms/28.5 ms | 0 V-90 V | Not touched | | | S25 |
| LC | 2 | - | 0.72-0.74 | 0.34-0.14 | 40 ms/200 ms | 0 V-70 V | Not touched | | | S26 |
| LC | 2 | 0.27-0.54 | - | - | 0.8 ms/40 ms | 0 V-50 V | Not touched | | | S27 |
| TESW | 1 | 0.58 | Not touched | | | | 55 | | 1.5 | S28 |
| TESW | 1 | 0.97 | Not touched | | | | 15 | | 8.2-12.4 | S29 |
| TESW | 1 | 0.52 | Not touched | | | | 32 | | 8.2-12.4 | S30 |
| TESW | 1 | 0.81 | Not touched | | | | 22.5 | | 8-12 | S31 |
| TESW | 1 | 0.85 | Not touched | | | | 25 | | 8.2-12.5 | S32 |
| TESW | 1 | 0.91 | Not touched | | | | 8 | | 8-12 | S33 |
| TESW | 1 | 0.76 | Not touched | | | | 37.5 | | 1 | S34 |

T_s_ ,Specular transmittance; T_t_ ,Total transmittance; H,Haze; t_on_,Response time; t_off_ ,Recovery time; T-D,Transition from transparent mode to dark mode; D-H,Transition from dark mode to haze mode; T-H,Transition from transparent mode to haze mode; EC, Electrochromic smart window; SPD,suspended particle devices ; LC,Liquid crystal material system; P-LC, Polymer-liquid crystal material; TESW,Transparent electromagnetic shielding window.

# References

S1 Deng, B. et al. An Ultrafast, Energy‐Efficient Electrochromic and Thermochromic Device for Smart Windows. **35** (2023).

S2 Zhao, Q. et al. Printing of WO_3_/ITO nanocomposite electrochromic smart windows. (2019).

S3 Zhang, S. et al. Amorphous and Porous Tungsten Oxide Films for Fast‐Switching Dual‐Band Electrochromic Smart Windows. **11** (2022).

S4 Ghosh, A., Norton, B. J. S. E. M. & Cells, S. Durability of switching behaviour after outdoor exposure for a suspended particle device switchable glazing. **163**, 178-184 (2017).

S5 Ghosh, A., Norton, B., Duffy, A. J. S. E. M. & Cells, S. First outdoor characterisation of a PV powered suspended particle device switchable glazing. **157**, 1-9 (2016).

S6 Yu, B., Ji, S. M., Kim, J.-H., Huh, J.-W. & Yoon, T.-H. J. J. o. I. D. Light shutter using dye-doped cholesteric liquid crystals with polymer network structure. **18**, 13 - 17 (2017).

S7 Xiao, L. et al. A temperature and electric field-responsive flexible smart film with full broadband optical modulation. **4**, 878-884 (2017).

S8 He, Z. et al. Silicon nanostructure-doped polymer/nematic liquid crystal composites for low voltage-driven smart windows. **33** (2021).

S9 Katariya-Jain, A., Deshmukh, R. R. J. J. o. P. & Solids, C. o. Effects of dye doping on electro-optical, thermo-electro-optical and dielectric properties of polymer dispersed liquid crystal films. **160**, 110363 (2022).

S10 Liang, X. et al. A roll-to-roll process for multi-responsive soft-matter composite films containing CsxWO_3_ nanorods for energy-efficient smart window applications. **2** 6, 319-325 (2017).

S11 Liang, X. et al. Dual-Band Modulation of Visible and Near-Infrared Light Transmittance in an All-Solution-Processed Hybrid Micro-Nano Composite Film. **9** 46, 40810-40819 (2017).

S12 Kim, M. et al. Fabrication of Microcapsules for Dye-Doped Polymer-Dispersed Liquid Crystal-Based Smart Windows. **7** 32, 17904-17909 (2015).

S13 Hu, X. et al. Stable and scalable smart window based on polymer stabilized liquid crystals. **137**, 48917 (2020).

S14 Zhang, Z. et al. Visible and infrared optical modulation of PSLC smart films doped with ATO nanoparticles. (2021).

S15 Ängskog, P., Bäckström, M., Samuelsson, C. & Kangashaka Vallhagen, B. J. I. T. o. E. C. Shielding Effectiveness and HPM Vulnerability of Energy-Saving Windows and Window Panes. **61**, 870-877 (2019).

S16 Zhan, Y., Lu, H., Jin, M. & Zhou, G. J. L. C. Electrohydrodynamic instabilities for smart window applications. **47**, 977 - 983 (2020).

S17 Konshina, E. A. & Shcherbinin, D. P. J. L. C. Study of dynamic light scattering in nematic liquid crystal and its optical, electrical and switching characteristics. **45**, 292 - 302 (2018).

S18 Zhan, Y., Schenning, A. P. H. J., Broer, D. J., Zhou, G. & Liu, D. J. A. F. M. Light‐Driven Electrohydrodynamic Instabilities in Liquid Crystals. **28** (2018).

S19 Huh, J.-W. et al. Ion-doped liquid-crystal cell with low opaque-state specular transmittance based on electro-hydrodynamic effect. **150**, 16-20 (2018).

S20 Huh, J.-W., Seo, J.-H., Oh, S.-W., Kim, S.-H. & Yoon, T.-H. J. J. o. M. L. Tristate switching of a liquid-crystal cell among initial transparent, haze-free dark, and high-haze dark states. (2019).

S21 Chen, C.-W., Brigeman, A. N., Ho, T.-J. & Khoo, I.-C. J. O. M. E. Normally transparent smart window based on electrically induced instability in dielectrically negative cholesteric liquid crystal. **8**, 691-697 (2018).

S22 Han, C.-H. & Oh, S.-W. J. D. A high-haze liquid crystal grating device with asymmetric anchoring energies. (2023).

S23 Huh, J.-W., Oh, S.-W., Seo, J.-H., Sohn, H.-j. & Yoon, T.-H. J. J. o. M. L. Filtering of yellow light in a liquid-crystal light shutter for higher color contrast and reduced glare. 114846 (2020).

S24 Li, C.-C. et al. Versatile energy-saving smart glass based on tristable cholesteric liquid crystals. **3**, 7601-7609 (2020).

S25 Madhuri, P. L., Shuddhodana, Judeh, Z. M. A., Abdulhalim, I. J. P. & Characterization, P. S. Cochleate‐Doped Liquid Crystal as Switchable Metamaterial Window Mediated by Molecular Orientation Modified Aggregation. **37** (2020).

S26 Abdulhalim, I., Madhuri, P. L., Diab, M. & Mokari, T. J. O. e. Novel easy to fabricate liquid crystal composite with potential for electrically or thermally controlled transparency windows. **27** 12, 17387-17401 (2019).

S27 Pappu, L. M., Martín-Palma, R. J., Martín-Adrados, B. & Abdulhalim, I. J. J. o. M. L. Voltage controlled scattering from porous silicon Mie-particles in liquid crystals. (2019).

S28 Kim, D., Kim, Y., Kim, J. W. J. M. & Design. Transparent and flexible film for shielding electromagnetic interference. **89**, 703-707 (2016).

S29 Hosseini, E., Arjmand, M., Sundararaj, U., Karan, K. J. A. a. m. & interfaces. Filler-Free Conducting Polymers as a New Class of Transparent Electromagnetic Interference Shields. (2020).

S30 Zhou, B. et al. Flexible MXene/Silver Nanowires-based Transparent Conductive Film with Electromagnetic Interference Shielding and Electro-photo-thermal Performances. (2020).

S31 Hu, M. et al. Flexible transparent PES/silver nanowires/PET sandwich-structured film for high-efficiency electromagnetic interference shielding. **28** 18, 7101-7106 (2012).

S32 Liang, X. et al. Room-Temperature Nanowelding of a Silver Nanowire Network Triggered by Hydrogen Chloride Vapor for Flexible Transparent Conductive Films. 9 46, 40857-40867 (2017).

S33 Zhang, H.-L., Xia, Y. & Gai, J. G. J. A. O. Ultrathin Active Layer for Transparent Electromagnetic Shielding Window. **3**, 2765 - 2772 (2018).

S34 Cheng, C.-M., Chen, Y.-H., Lin, S.-Y., Chao, S. D. & Tsai, S.-F. J. T. Study on Shielding Effectiveness of High Transmittance Coating Film Glasses against Electromagnetic Pulse. (2023).
